# Pupil diameter tracked during motor adaptation in humans

**DOI:** 10.1152/jn.00021.2022

**Published:** 2022-10-05

**Authors:** Atsushi Yokoi, Jeffrey Weiler

**Affiliations:** ^1^Center for Information and Neural Networks, Advanced ICT Research Institute, National Institute of Information and Communications Technology, Suita, Japan; ^2^Graduate School of Frontier Biosciences, Osaka University, Suita, Japan; ^3^Schulich School of Medicine and Dentistry, Western University, London Ontario, Canada; ^4^The Gray Centre for Mobility and Activity, Parkwood Institute, London, Ontario, Canada; ^5^The Brain and Mind Institute, Western University, London, Ontario, Canada; ^6^Department of Physiology and Pharmacology, Western University, London, Ontario, Canada

**Keywords:** force field, motor learning, pupil dilation, reaching

## Abstract

Pupil diameter, under constant illumination, is known to reflect individuals’ internal states, such as surprise about observation and environmental uncertainty. Despite the growing use of pupillometry in cognitive learning studies as an additional measure for examining internal states, few studies have used pupillometry in human motor learning studies. Here, we provide the first detailed characterization of pupil diameter changes in a short-term reach adaptation paradigm. We measured pupil changes in 121 human participants while they adapted to abrupt, gradual, or switching force field conditions. Sudden increases in movement error caused by the introduction/reversal of the force field resulted in strong phasic pupil dilation during movement accompanied by a transient increase in tonic premovement baseline pupil diameter in subsequent trials. In contrast, pupil responses were reduced when the force field was gradually introduced, indicating that large, unexpected errors drove the changes in pupil responses. Interestingly, however, error-induced pupil responses gradually became insensitive after experiencing multiple force field reversals. We also found an association between baseline pupil diameter and incidental knowledge of the gradually introduced perturbation. Finally, in all experiments, we found a strong co-occurrence of larger baseline pupil diameter with slower reaction and movement times after each rest break. Collectively, these results suggest that tonic baseline pupil diameter reflects one’s belief about environmental uncertainty, whereas phasic pupil dilation during movement reflects surprise about a sensory outcome (i.e., movement error), and both effects are modulated by novelty. Our results provide a new approach for nonverbally assessing participants’ internal states during motor learning.

**NEW & NOTEWORTHY** Pupil diameter is known as a noninvasive window into individuals' internal states. Despite the growing use of pupillometry in cognitive learning studies, it receives little attention in motor learning studies. Here, we characterized the pupil responses in a short-term reach adaptation paradigm by measuring pupil diameter of human participants while they adapted to abrupt, gradual, or switching force field conditions. Our results demonstrate how surprise and uncertainty reflected in pupil diameter develop during motor adaptation.

## Graphical abstract

**Figure:**
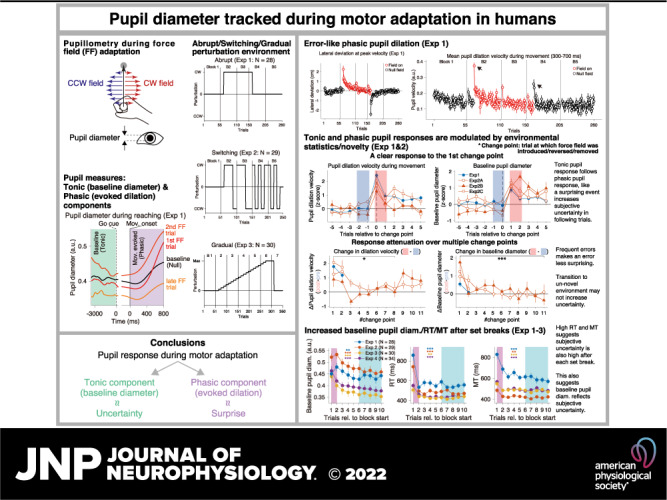

## INTRODUCTION

Motor learning, as a process of correcting movements to achieve a goal, is a complex mixture of multiple processes (1–3). For instance, riding a bicycle initially requires substantial effort to control the handlebars and pedals while balancing, but these conscious efforts are eventually taken over by more automatic and implicit control processes. Studies using arm reaching posit that motor adaptation to a novel dynamic/kinematic environment consists of multiple processes, which are often contrasted as conscious/explicit and automatic/implicit components (4–6), each separately depending on prefrontal and cerebellar function (2, 7). Indeed, human functional imaging studies have revealed that during the acquisition of new motor skills, prefrontal and hippocampal areas are particularly active in the earliest stage of learning. In later stages, the cortical motor and parietal areas, as well as subcortical regions, become more active (8–11). A more recent study has further proposed a view that networks of these brain areas, rather than in isolation, underly the dual processes of motor adaptation (12). However, the precise cognitive processes represented by early activation of the “more cognitive network” and the ways in which these processes evolve at finer temporal scales remain unclear.

Pupil diameter under constant illumination is known to reflect a variety of internal cognitive states of individuals performing cognitive or simple motor tasks. Task-evoked changes reflected in phasic pupil dilation have been associated with (unsigned) prediction error or surprise about observations (13–19), mental/physical effort (20–22), and motivational vigor (23). Relatively slow changes in tonic baseline diameter have often been associated with arousal/vigilance (24–29), the tendency of the exploration/exploitation trade-off (30, 31), and more recently, subjective uncertainty about the environment (13–18, 32). Although these constructs have different names, they are closely related to each other in terms of their dynamics. For instance, a surprising observation (i.e., a large deviation from an expectation) may imply a change in the environment leading to a (transient) increase in subjective environmental uncertainty. In an uncertain situation, an animal may need to make more mental/physical effort to find a better solution (i.e., exploration), which may recruit increased arousal/vigilance/vigor. As the animal adapts to the new environment, surprise about observations and other variables gradually returns to the average level. Thus, surprise and uncertainty appear to be essential for interpreting pupil responses in various tasks.

More importantly, surprise and uncertainty are believed to play a crucial role in learning behavior by dynamically adjusting learning rate, especially in reward-based learning, including conditioning (33) and choice decision-making (16, 34–36). Thus, these constructs could similarly affect motor learning. However, there have been few attempts to assess the trial-by-trial changes in pupil diameter during human motor learning. In the current study, to characterize pupil responses and the types of information they reflect in a commonly used motor learning paradigm, we ran a series of experiments in which we simultaneously tracked pupil diameter during short-term force field adaptation in a reaching paradigm (37). In our experiments, we recruited 121 participants who performed reaching movements under the presence of a velocity-dependent force field that was introduced either abruptly (*n* = 28), abruptly and then reversed multiple times (*n* = 29), or gradually (*n* = 30 and 34). Our data suggest that the tonic baseline pupil diameter reflects participants’ belief about environmental uncertainty, whereas the phasic pupil dilation during movement reflects surprise about a sensory outcome (i.e., movement error), and both are modulated by the novelty of perturbation. The current study thus provides an interesting new approach for nonverbally assessing participants’ cognitive states during motor learning.

## MATERIALS AND METHODS

### Participants

We recruited 28, 30, 30, and 35 right-handed participants with no history of neurological disorders for *experiments 1, 2, 3*, and *4*, respectively (76 males, 47 females; age: 19–37 yr). Participants provided written informed consent before taking part in the study. All of the experimental protocols were approved by the ethical committees of the University of Western Ontario (*experiment 1*), Osaka University (*experiment 2*), and the Center for Information and Neural Networks (*experiments 3* and *4*). The sample sizes were selected on the basis of previous studies focusing on pupil size as a main variable of interest (13, 16, 38, 39).

### Experimental Settings

#### General settings.

Participants were instructed to perform a straight center-out reaching movement from a starting position to a goal target in a two-dimensional plane while holding the handle of a robotic manipulandum. The starting position (white circle, 1.6-cm diameter), the target (gray circle, 1.6-cm diameter), and participants’ current hand position (white dot cursor, 0.5-cm diameter) were displayed on liquid-crystal display (LCD) monitors. The target was located either 12 cm (*exp 1*) or 10 cm (*exp 2/3*) directly in front of the starting position. Throughout the task, we monitored the participants’ eye gaze and pupil diameter using eye trackers (EyeLink 1000, SR Research Ltd., ON, Canada). To reduce the impact of unnecessary eye movements on pupillometry measurement, participants were required to maintain fixation on the center of the goal target. Before the main experimental session, participants underwent a familiarization session with continuous visual feedback of their current hand position (i.e., the cursor). In the main experimental sessions, online visual feedback of the cursor during movement was removed and only terminal feedback was provided at movement offset. For both the familiarization and main session, participants were instructed to aim directly at the target. The colors used for the start position (gray), target (gray/green), background disk (pale blue), and endpoint feedback (magenta) were adjusted to ensure that they were approximately isoluminant (details provided in Table 1).

**Table 1.** Luminance value measurements for display colors

	*Exp 1*	*Exps 2–4*
Black	0.6	0.4
White	178	249
Gray	80	107
Green	80	106
Magenta	80	108
Pale blue	80	105

Measured by CA-100 plus color analyzer (Konica Minolta Sensing Americas, Inc.) for *exp 1* and by SR-3AR Spectroradiometer (Topcon Technohouse, Corp., Tokyo, Japan) for *exps 2–4*. Units are in cd/m^2^.

At the start of each trial, the robot’s handle automatically moved the participants’ hand into the starting position. During a trial, participants maintained the cursor at the start location for 1 s while maintaining fixation on the center of the target. Following this period, we measured participants’ pupil diameter for a variable duration (3–11 s). The start position then changed to green, informing the participants to initiate reaching. Movement onset was defined as the time point at which the hand movement velocity first crossed a threshold (3.5 cm/s). Similarly, movement offset was defined as the time point at which the hand movement velocity first dropped below the threshold since the movement onset. At the completion of a reaching movement, endpoint feedback was provided by a magenta cursor (0.5-cm diameter) for 1,000 ms.

We introduced a velocity-dependent curl force field (40) to establish the relationship between the pupil response and the motor adaptation. The force field was applied according to the following equation:

(*1*) $$\left[\begin{array}{c} f_{x}\\ f_{y} \end{array}\right]=\left[\begin{array}{cc} 0 & B\\ -B & 0 \end{array}\right]\left[\begin{array}{c} v_{x}\\ v_{y} \end{array}\right]$$

where *f_x_* and *f_y_* are the force applied to the handle (N), and *v_x_* and *v_y_* are the velocities of the handle (m/s) for the *x*- and *y*-directions, respectively. For the clockwise (CW) force field, the viscosity coefficient B [N/(ms^−1^)] had positive values, and for the counter-clockwise (CCW) field, B had negative values. To quantify adaptation to the force field, we occasionally introduced “channel” trials, in which the handle motion was constrained to a straight path between the home position and the target by a simulated damper and spring (41), to measure the force applied to the channel.

**Figure 1.**
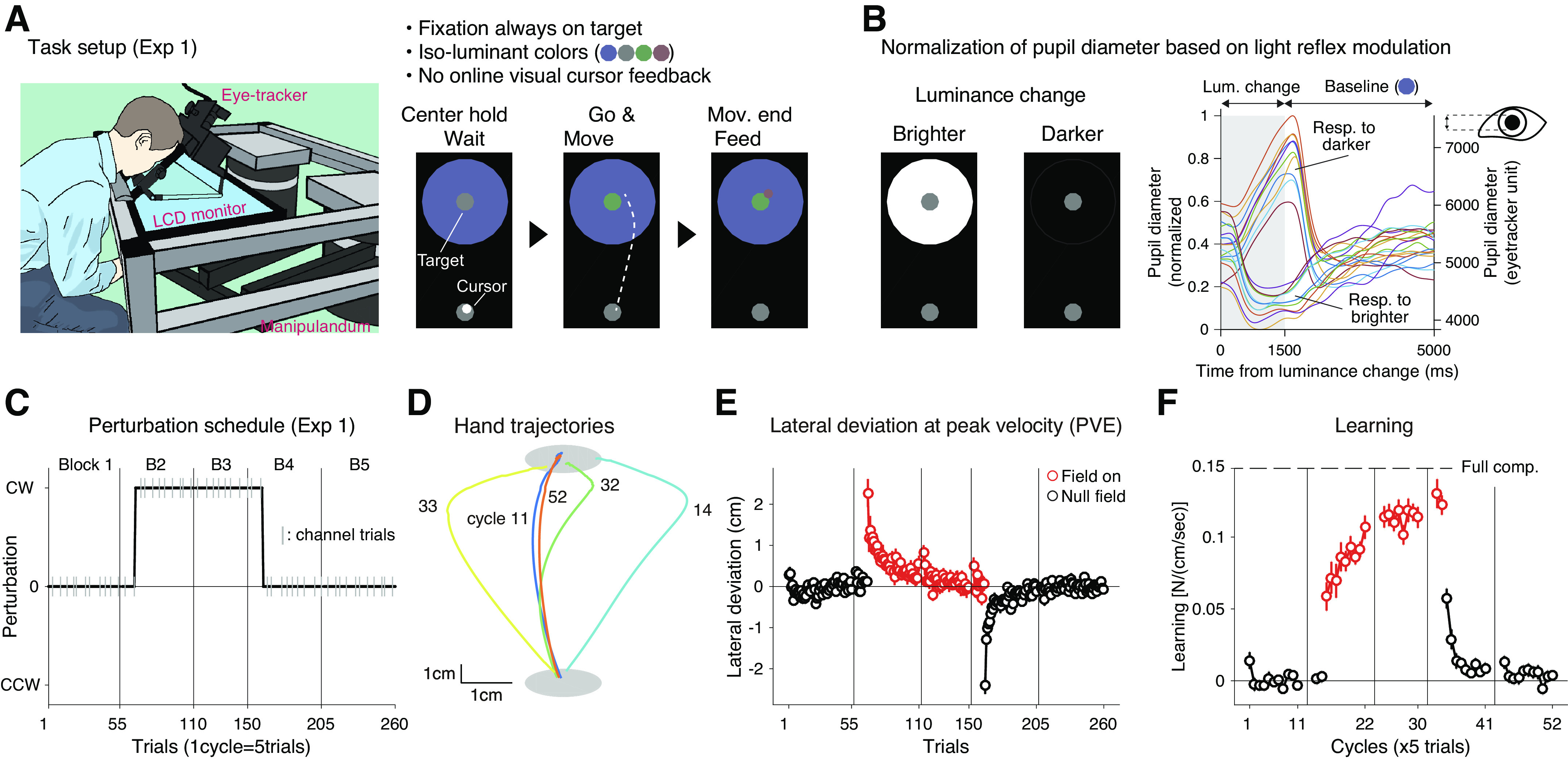
Pupillometry during force field motor adaptation (*experiment 1*). *A*, *left*: experimental setup used in *exp 1* (*n* = 28 subjects). *Right*: schematics for a single trial. Participants were required to fixate on the center of the target. *B*: pupil diameter data were individually normalized using modulation size of pupil light reflex induced by bright/dark background color change (*left*). Example time courses of pupillary responses in one participant are shown on the *right*. Different colors indicate individual trials. *C*: the perturbation schedule for *exp 1*. The manipulandum applied the clockwise (CW) force field unexpectedly for the participants in the second block. The gray vertical lines indicate set breaks. The short gray vertical lines represent the force channel trial where learning was quantified. *D*: average hand trajectories of participants who learned with the CW force field. The cycle numbers (11, 14, 32, 33, and 52) correspond to baseline, early perturbation, late perturbation, early washout, and late washout trials. *E*: mean lateral hand deviation at the peak tangential handle velocity (PVE). Positive values correspond to rightward deviation. *F*: mean learning index measured in the channel trials (once in a cycle). Learning index was the lateral force to the channel at the time of peak velocity divided by the peak velocity (i.e., viscosity). The red dots represent data for perturbation trials (*E* and *F*). Error bars correspond to the standard error of the mean (SEM) across the participants (*E* and *F*).

To make interparticipant comparisons of pupil diameter interpretable (i.e., for *exps 3* and *4*), we additionally measured reference physiological response amplitudes of pupil diameter within each participant by changing the background color of the display from light blue to white (higher luminance) or black (lower luminance) (Table 1) to elicit a pupillary light reflex. These measured pupil limits were used for the within-individual normalization of pupil diameter data (Fig. 1B; see Supplementary Note and Supplemental Fig. S1 for more detail).

#### Experiment 1.

*Experiment 1* was conducted at the Brain and Mind Institute, University of Western Ontario (Ontario, Canada). The self-reported right-handed participants (*n* = 28; 13 males, 15 females; age: 24.3 ± 4.5) sat on a height-adjustable chair and held the handle of a robotic manipulandum (1,000 Hz control rate) (42). The position of the handle was represented as a cursor (white dot) on an LCD monitor (60 Hz update rate) placed directly above the handle to prevent the participant from seeing their hand. The starting position (white circle), goal target (gray circle), and background (light blue circle, 15 cm diameter), to prevent sharp luminance changes around the target, were also presented on the display throughout the task (Fig. 1*A*). The eye tracker was mounted on the display to monitor participants’ eye gaze and pupil diameter (Fig. 1*A*). The approximate distance between participants’ eyes and the center of the monitor was 16 cm.

An experimental session consisted of five blocks of trials (59 trials per block, except for the fourth block, which consisted of 44 trials). There were short breaks (up to 1 min) inserted between the blocks. On each block, the first and the last two trials were used to measure the simple pupil light reflex of the participants. In each of these “light reflex” trials, the LCD screen was suddenly turned either black or white for 1,500 ms. The pupil response was measured during and up to 3,500 ms after the termination of the black/white color stimulus. The response strengths (i.e., trough for constriction and peak for dilation) were averaged over the blocks and used to normalize the individual pupil diameter during the main task. Following the initial “light reflex” trials, participants performed 55 trials of the center-out reaching task. On each trial, after confirming stable eye fixation on the goal target (1,000 ms of fixation without any blink) and the cursor staying within the home position, the goal target turned green after a variable delay (1,000–1,500 ms) cueing the reaching movement. To prevent possible predictive/reflexive eye movement and/or pupil dilation because of a moving cursor, visual feedback of the cursor was removed during movement and participants were instructed to maintain fixation on the center of the goal target. Terminal endpoint feedback was provided with a near-isoluminant magenta cursor for 1,000 ms when the cursor speed was less than 1 cm/s. After the feedback period, the manipulandum handle automatically returned to the home position and the next trial started.

*Experiment 1* used a typical null-force-null paradigm. The force field was introduced at the 11th trial of the second block and removed at the 11th trial of the fourth block (Fig. 1*C*). Participants were not informed regarding the trial on which the perturbation would be introduced or removed in advance. Twenty participants were presented with the CW force field (*B* = 0.15 in *Eq*. *1*) and the remaining eight participants were presented with the CCW force field (*B* = −0.15). To quantify adaptation to the force field, we interleaved the channel trials in 20% of the trials. The channel stiffness and viscosity were 7,000 (N/m) and 30 [N/(ms^−1^)], respectively.

#### Experiments 2, 3, and 4.

*Experiments 2–4* were conducted at the Osaka University/Center for Information and Neural Networks (Suita, Japan). Participants sat on a height-adjustable chair and held the handle of a robotic manipulandum (Phantom Premium HF 1.5, 3 D Systems, Inc.). The force field strength was set to *B* = 0.12 (CW) or −0.12 (CCW). The channel stiffness and viscosity were 2,500 (N/m) and 25 [N/(ms^−1^)], respectively. The position of the handle was visually displayed on a vertically placed LCD monitor (60 Hz update rate) as a cursor (white dot). A starting position (gray circle), a goal target (filled gray circle), and a background disk (light blue disk, 21.6 cm diameter) that was used to prevent sharp luminance changes around the target were also presented on the display throughout the task (Fig. 3*A*). The colors (gray, green, light blue, and magenta) were adjusted to be approximately isoluminant (Table 1). A desktop eye tracker (Eyelink 1000, SR Research, ON, Canada) was placed under the LCD display to monitor participants’ eye gaze and pupil diameter (Fig. 3*A*). The approximate distance between participants’ eyes and the center of the monitor was 35 cm for *exp 2* and 44 cm for *exp 3* and *4*. We assessed participants’ handedness using a Japanese-translated version of the FLANDERS handedness questionnaire (43, 44), which ranges from +10 (perfect right-hander) to −10 (perfect left-hander).

##### Experiment 2.

In *exp 2*, 30 right-handed individuals participated. Participants were divided into three subgroups *A–C* (10 participants each). One participant assigned in *exp 2C* did not complete the whole experimental session because of frequent blinks during an experimental trial. As a result, the data from 29 participants (24 males, 5 females; age: 22.3 ± 2.7; handedness score: 9.9 ± 0.3) were analyzed (10 for *exp 2A*, 10 for *exp 2B*, and 9 for *exp 2C*). All three experiments consisted of five blocks of 50 reaching trials with short breaks (∼1 min) between the blocks. Similar to *exp 1*, in each block, we added two trials of light-reflex measurements before and after the reaching trials. The perturbation schedules were characterized with sudden reversals in force field direction, as summarized in Fig. 3*B*. For *exp 2A*, CW force was applied on trials = {61:89, 101:129, 151:159, 170:179, 190:209, 220:229}, and CCW force was applied on trials = {90:100, 130:150, 160:169, 180:189, 210:219, 230:239}. For *exp 2B*, CW force was applied on trials = {70:79, 90:109, 120:129, 140:189, 201:229}, and CCW force was applied on trials = {61:69, 80:89, 110:119, 130:139, 190:200, 230:239}. For *exp 2C*, CW force was applied on trials = {61:159}, and CCW force was applied on trials = {160:170}. For all groups (*exp 2A* through *2C*), channel trials were randomly interspersed in 20% of trials, except that for *exp 2C*, channel trials were repeated from the 171st trial to the 250th (last) trial.

##### Experiment 3.

In *exp 3*, 30 right-handed individuals participated (18 males and 12 females; age: 22.3 ± 2.3 yr; handedness score: 9.2 ± 3.1). In this experiment, the force field was gradually introduced over the course of seven blocks (50 trials in each block). The force field was first introduced on the 16th trial in the second block and incrementally increased by 5% of the full-strength (*B* = 0.12) after every 11 trials, until it reached the full-strength (Fig. 5*A*). In *block 7*, the force was abruptly removed on the 11th trial (Fig. 5*A*). Channel trials were randomly interspersed in 20% of trials. Short breaks of 1 min were inserted between each block, except that it was 3 min after the third block of trials. After the last block, the participants went through an additional block consisting only of light-reflex trials. The dark and bright conditions were alternated for five trials each (total of 10 trials). Duration of luminance change was set to 3,000 ms, and pupil response was measured for additional 3,000 ms after the termination of luminance change. Half the participants adapted to the CW force field, and the rest of the participants adapted to the CCW force field. The force and kinematic data were sign-flipped for the CCW participants. After the experiment, the participants in *exp 3* answered a questionnaire asking if they realized any force perturbation. The questions included *1*) whether they felt any load in any phase of the experiment (yes/no); *2*) which direction of load they felt during the experiment (1: forward; 2: backward; 3: rightward; 4: leftward; 5: other, multiple answers allowed); and *3*) in which of the seven blocks they felt the above load (1–5, multiple answers allowed, for each block). In the current experiment, the data for question (3) were analyzed after binarizing the answers into whether they felt any load (0/1) for each block.

##### Experiment 4.

We also ran a variant of *exp 3* (*exp 4*) with 35 additional participants. In this experiment, *block 7* consisted of 70 trials in which the channel trial was repeated from 21st through the 70th trial, instead of force removal. Otherwise, all the protocols were the same as *exp 3*. The data from one participant were excluded from the analysis because of a technical failure in collecting pupil calibration data. As a result, the data from the remaining 34 participants were analyzed (20 males and 14 females; age: 22.2 ± 1.6 yr; handedness score: 9.9 ± 0.9). Nineteen participants adapted to the CW force field, and the rest of the participants adapted to the CCW force field. Of these 34 participants, 32 individuals answered the poststudy questionnaire about the force perturbation.

### Data Analysis

The data were sampled at 200 Hz. All analyses were conducted using custom-written codes with MATLAB R2015b (Mathworks, Natick, MA).

For all of the experiments, the data from the manipulanda (x-y positions, x-y velocities, and x-y command forces for the manipulandum handle) were smoothed with a Gaussian kernel of 35 ms full width half maximum (FWHM). Reach onset was defined by the time at which the tangential hand velocity first exceeded 10% of its peak value. Reach offset was defined as the time at which the tangential hand velocity first dropped below the threshold determined for reach onset of that trial. To assess movement errors on each reach, we computed peak velocity error (PVE) and endpoint error (EPE). PVE was defined as perpendicular hand displacement at the time of peak tangential hand velocity with respect to a straight line from the home position to the target. EPE was defined as the distance between the hand and the target positions at movement offset. As an index of motor learning, we used the x-force values at the time of peak tangential hand velocity divided by the peak velocity during the channel trials (45). For *exps 1, 3, and 4*, the PVE and learning index were sign-flipped for participants who learned with the CCW force field before taking the group average.

For all the experiments, the eye position and pupil data (x-y point-of-gaze position and pupil diameter) were analyzed in the following way. First, we discarded 100 ms of point-of-gaze position and pupil data before a blink event and 150 ms of point-of-gaze position and pupil data after a blink event. We then interpolated over the discarded data using a piecewise cubic Hermite interpolating polynomial (Matlab *interp1.m* function with “pchip” option). After the interpolation, the eye position data were smoothed with Gaussian kernel with 35 ms FWHM. For the detection of saccade events, the unsmoothed eye position data were also smoothed with a second-order Savitzky-Golay filter with a frame length of 55 ms (*sgolayfilt.m* function). The eye velocities were then calculated by the numerical derivative of the eye positions (*diff.m* function). We used 30°/s as the velocity threshold for saccade detection. Because the eye positions were defined in the display coordinates, we transformed them to visual angle coordinates before the saccade detection.

**Figure 2.**
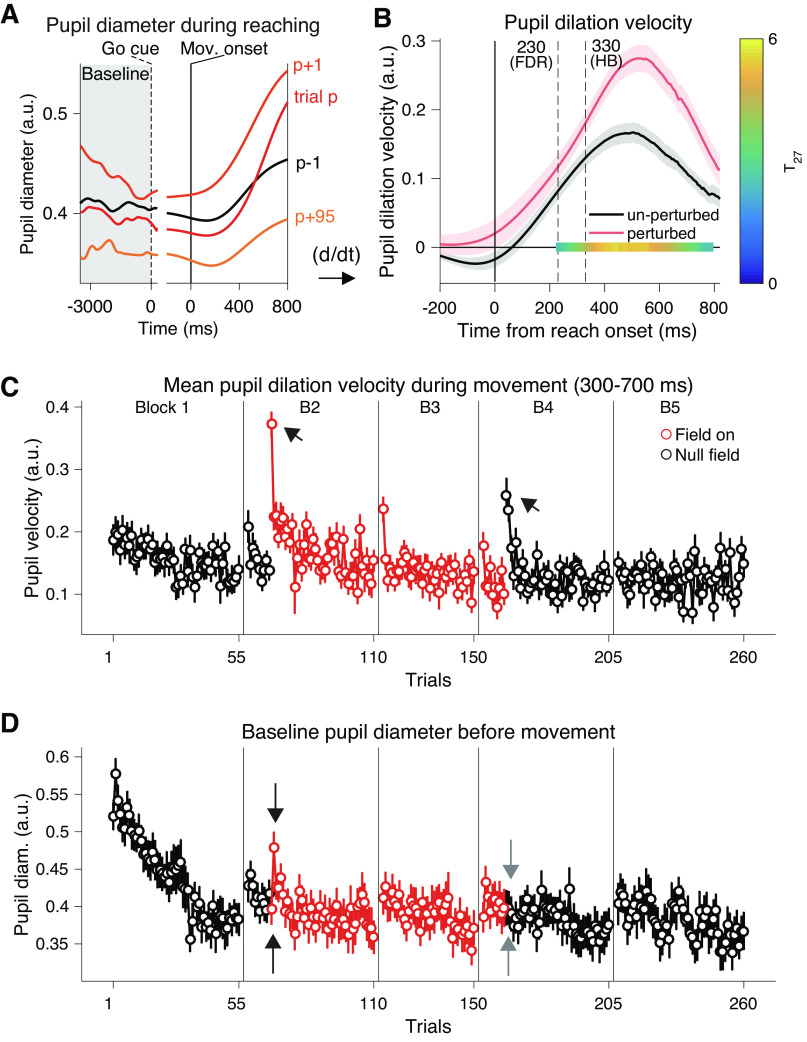
Pupil responses to abruptly introduced force field (*experiment 1*). *A*: averaged pupil diameter time series across participants during *exp 1*, aligned either to the go cue or reach onset. In general, the pupil diameter consisted of tonic (premovement baseline diameter) and phasic (movement-evoked dilation) components. On a sudden perturbation trial (indicated as trial *p*), the pupil was strongly dilated (phasic response; see also arrows in *C*). On the following trial (trial *p* + 1), pupil diameter showed a higher baseline value (tonic response; see also arrows in *D*). On the later trial (trial *p* + 95), phasic pupil dilation returned to the baseline level and tonic premovement diameter decreased below baseline level. *B*: the phasic pupil response to the force perturbation; the first time-derivative (= velocity) of the pupil diameter time series (*A*) averaged over the trials before the force field was introduced (unperturbed; black trace) or the first five perturbation trials at the second block (perturbed; red trace). The areas around the traces represent the SEM across the participants. The vertical dashed lines indicate the first time point of significant difference assessed by running paired *t* tests (two-sided) corrected by either the Holm–Bonferroni method (HB; *P* < 0.05) or the Benjamini–Hochberg method controlling for false discovery rate (FDR; *q* < 0.05). A significant (*P* < 0.05) cluster assessed by cluster-based permutation test is also shown on the *y* = 0 line, with color mapping representing *t* values for comparison (T27; the color bar on the right). *C*: trial-by-trial changes in the pupil dilation velocity averaged between 300 and 700 ms from movement onset. *D*: trial-by-trial changes in the baseline pupil diameter (mean pupil diameter during the hold and wait period; see *A*). The red circles represent data for perturbation trials (*C* and *D*). Error bars correspond to the SEM across participants (*C* and *D*). The arrows in *C* indicate the sharp increases in mean pupil dilation velocity at the first introduction/removal of the force field. The arrows in *D* indicate the first two perturbation trials (upward: the first trial, downward: the second trial), and the first two trials since force removal (upward: the first trial, downward: the second trial).

To remove high-frequency noise, the pupil data were smoothed with a Gaussian kernel with 235 ms FWHM. Importantly, we individually normalized the pupil diameter data relative to the minima and maxima of the pupil diameter data measured during the light reflex trials. We also calculated the pupil dilation velocity through the numerical derivative (*diff.m* function) of the normalized pupil diameter data. The trial-by-trial summaries of these pupil-related variables were defined as follows. The baseline pupil diameter was defined as the average pupil diameter during the waiting period before the onset of the go cue. The mean pupil dilation velocity at each trial was defined as the average of pupil dilation velocity during the period from 300 to 700 ms from movement onset. These periods were defined in a post hoc manner to maximally reflect the effect of experimental manipulation (i.e., force perturbation) and roughly corresponded to the periods of *P* < 0.001 (uncorrected) for the comparison between the baseline versus the first five perturbed trials (e.g., Fig. 2*B*). Pupil dilation velocity data for trials in which a saccade was detected during the movement period were excluded from the analysis. The percentages of excluded trials were 16.5 ± 12.9% (*exp 1*), 15.5 ± 15.2% (*exp 2*), 5.3 ± 4.5% (*exp 3*), and 10.5 ± 8.8% (*exp 4*).

### Statistical Analysis

#### Time-series comparison (exp 1 and 2).

We assessed changes in the pupil velocity time series between unperturbed (average of the first block data) versus perturbed (average over the first five perturbed trials) trials using group-wise two-sided paired *t* tests applied at each time frame. Uncorrected *P* values were reported. Correction for multiple comparisons (no. of time frames since movement onset) was applied using the Holm–Bonferroni method (46) with a family-wise error rate of *P* = 0.05 and the Benjamini–Hochberg method (47) with a false discovery rate of *q* = 0.05. For a complementary analysis, we also used the cluster-mass permutation test (48) implemented as ‘*permutest.m’* for MATLAB (49) with a significance level of *P* = 0.05 using a cluster-thresholding *P* value of 0.01. However, it should be noted that the cluster-based permutation test does not establish the significance of effect latency (50).

#### Evaluation of pupil dilation at force removal (exp 1).

We fitted the linear mixed-effects model to the pairs of pupil dilation velocity and absolute movement error (lateral deviation) data for baseline (averaged over 45th–55th trials) and for the first force field trial. The model contained fixed intercept and slope for error and their random effects over participants. By feeding the individual error data for the first washout trial to the trained model, we generated the predicted pupil dilation data and compared them with the actual pupil dilation data (two-sided paired *t* test).

#### Test for faster washout (exp 1).

We tested whether washout is faster than the initial learning by both model-based and model-free ways. For the model-based analysis, we first trained the two-state model (51) with the learning index data up to the last learning trial (training data) with 1,000 bootstrap resampling over participants. Then, we compared the predicted bootstrap distribution of learning indices for the washout phase and the actual group-average data (test data). For the model-free analysis, we calculated the average trial numbers that were needed to achieve either 40%, 50%, 60%, 70%, or 80% change in behavioral measure (PVE or learning index) and compared them between the learning versus washout phases. For this analysis, data for washout phase were flipped relative to their plateau and transformed into %-change index. To account for the random fluctuation in the data, we defined the point of *x*% change as the trial/cycle at which the data was above *x* for *n* times in total (*n* was set to 10 for kinematic error and 3 for learning index).

#### Effect of repetitive change points (exp 2).

We assessed the effect of participants’ repetitive experience of the change points by fitting a general linear mixed-effects model (*‘fitlme.m’* function) to the individually z-scored response data around the change points (baseline pupil diameter, pupil dilation velocity). Here, a change point refers a trial with a sudden introduction, reversal, or removal of force field. All models included a fixed intercept and random intercepts for participants and groups (*exps 2A, 2B, and 2C*) and fixed and random effects of the perturbation types (CW and CCW). As effects of interest, fixed and random effects for the number of change points (#cps) and the length of previous environment preceding change points (lcp) were also included. To account for the trivial effect of error magnitude, we additionally included the absolute error term: trajectory error and endpoint error at the immediately previous trial of change points for the baseline pupil data and trajectory error at change points for the pupil dilation velocity data. The subject, group, and perturbation types were treated as categorical variables. The importance of the random effect of #cp was assessed using the likelihood-ratio test (LRT) between the full model and an alternative model lacking the random slope term. The models were fitted to the data using the restricted maximum likelihood method (ReML) with random starting values. We evaluated the *P* value of the estimated fixed-effect slope for the #cp and lcp terms to test whether the effect of change in #cp and lcp were statistically significant. The significance level was set to 0.05.

#### Subgroup comparison (exps 3 and 4).

The data for all 30 participants in *exp 3* and 32 participants in *exp 4* who went through the poststudy questionnaire about perturbation were analyzed. The report data consisted of a binary vector of length 7 for each participant (absent/present for each of the seven blocks). In perfect case, this would yield [0, 1, 1, 1, 1, 1, 1]. We first split the data into two subgroups based on the median of the total report score (i.e., sum of the report vector, values ranging from 0 to 7). As the total report score took discrete values, this splitting yielded slightly uneven numbers for low- versus high-memory groups (*n* = 14 vs. 16 for *exp 3* and *n* = 14 vs. 18 for *exp 4*). The block-wise difference in the score (i.e., number of “present”) between each subgroup was assessed using a *χ*^2^ test at each block. We then compared the pupil and behavior data between the subgroups in the following way. Data were first averaged within each cycle (five-trial bins), and a linear mixed-effects model was fit to the data. For *exp 3*, the data in the washout phase was not included in the analysis for pupil dilation, movement error, and learning index as it induced a discontinuous change in these measures. The model contained a fixed intercept, a random intercept regarding subjects, a fixed effect for cycle number within each block, random effects for cycle number regarding subject and block, and a fixed interaction between block and subgroup as an effect of interest. A significant difference between the subgroups was assessed by the significance of this interaction term for each block. The cycle number was treated as a continuous variable, and other variables were treated as categorical. The models were fit to the data with the ReML method with random starting values. We used the Holm–Bonferroni method (46) for correction for multiple comparisons (i.e., blocks) to maintain the family-wise error rate at *P* = 0.05. For less-stringent correction, we also used the Benjamini–Hochberg method (47) to maintain the false discovery rate at *q* = 0.05.

#### Effect of set break (block novelty).

To test the effect of starting new blocks and avoiding the effect of perturbation, we first selected the blocks in each experiment in which perturbation was either absent or very weak for the initial 10 trials (*blocks 1, 2*, and *5* for *exp 1*, *1* and *2* for *exp 2*, and *1–4* for *exps 3* and *4*) and averaged the data within each trial defined relative to block initiation. We then compared the average of the initial two trials (early) versus the 6th–10th trials (late) for baseline pupil diameter, pupil dilation, reaction time, and movement time, using a two-sided paired *t* test for each experiment.

### Data and Code Availability

The data and the custom-written Matlab codes used for the analysis are available at https://github.com/ayokoi/pupilMotor01.

## RESULTS

### Pupillometry During Simple Force Field Adaptation Paradigm: Error-Like, but Not Purely Error-Driven Pupil Responses

To obtain initial insights about pupil diameter changes during a typical motor adaptation paradigm, we monitored 28 participants’ eyes while they performed a reaching task with a force field perturbation (*exp 1*; Fig. 1, *A* and *C*). To minimize measurement noise in pupillometry, we instructed the participants to keep their gaze fixated on the target and to refrain from blinking while they performed reaching movements. The visual cursor feedback was occluded during reaching. To minimize brightness-induced changes in pupil size, visual stimuli were isoluminant with respect to the background (Fig. 1*A*). Participants were instructed to aim and reach directly at the target. Participants showed a typical behavioral signature of force field adaptation. A sudden introduction of the perturbation force disturbed the smooth, relatively straight hand trajectory (*cycle 11*, Fig. 1*D*) resulting in a large lateral deviation (*cycle 14*, Fig. 1*D*). The lateral deviations rapidly decreased with repeated reaches made under the perturbation (*cycle 32*, Fig. 1, *D* and *E*), and the sudden removal of the force perturbation resulted in a large hand deviation toward the opposite direction (*cycle 33*, Fig. 1, *C* and *D*), a signature of motor adaptation known as an aftereffect. With additional trials, the trajectory (and movement errors) returned to a near-baseline level (*cycle 52*, Fig. 1, *D* and *E*). The learning quantified in the force channel trials also showed a typical learning curve (Fig. 1*F*). How do participants’ pupils respond in this typical motor adaptation situation?

The pupil responses during a task are frequently categorized into two aspects: a rapid change during a trial in response to task factors (phasic component) and a slow change in pretrial baseline diameter (tonic component) (15, 16, 52, 53). Here, following this convention, we also present our results in terms of the phasic and tonic pupil responses.

The pupil typically showed phasic dilation during movement in the null-field trials (Fig. 2*A*, *p*–1). When the perturbation was unexpectedly applied, the pupil exhibited additional dilation (Fig. 2*A*, trial *p*) and an increase in baseline diameter (Fig. 2*A*, *p* + 1). After sufficient number of trials, the pupil response returned to near-baseline level (Fig. 2*A*, *p* + 95). Analysis of the time derivative of the pupil dilation (pupil dilation velocity) revealed that this perturbation-evoked pupil dilation started ∼300 ms after movement onset and lasted until ∼700 ms after movement onset (Fig. 2*B*). The trial-by-trial change in the pupil dilation velocity averaged over this window (we will simply refer to this as pupil dilation) showed a sharp rise upon the introduction of the force field and gradual decline as the participants adapted to the force field and the movement error decreased (Fig. 2*C* and 1*E*). This indicates that, in the current paradigm, the phasic pupil dilation during reaching did not simply reflect physical effort, as it decreased despite the increase in lateral force exerted by the participants (Fig. 1*F* and 2*C*). Faced with the sudden removal of the force field and the resultant aftereffect, the phasic pupil dilation once again increased (Fig. 2*C*; *block 4*). The correlation between the trial-by-trial change of phasic pupil dilation and that of absolute movement error for the group-averaged data was highly significant (*r* = 0.64; *P* = 8.97 × 10^31^; bootstrap 95% confidence interval: [0.43, 0.80]), suggesting that the phasic pupil dilation may reflect error size. However, the size of error-induced pupil dilation at the first washout trial was smaller than what would be expected given its error size. We fit a linear mixed-effects model to the pupil dilation with error size as an independent variable. We used the baseline and the first force field trial data (Supplemental Fig. S2*A*) for training the model and then predicted the pupil response using the error size data for the first washout trial. The actual pupil response was significantly smaller than the predicted response (*t*_23_ = −2.53, *P* = 0.019). Thus, although phasic pupil dilation is responsive to sudden change in movement error, it may not reflect the error itself.

We also analyzed trial-by-trial change in the tonic baseline pupil diameter averaged over premovement period (Fig. 2, *A* and *D*). The initially large baseline pupil diameter showed rapid decrease during the first block (Fig. 2*D*), a pattern that has been commonly observed (54–57) and thought as reflecting a mixture of processes related to the familiarization to the experiment setup (e.g., uncertainty, mental effort, exploration) or general decline in arousal/vigilance. It is also noteworthy that the tonic baseline pupil diameter was larger at the beginning of a new block (Fig. 2*D*). We will later provide more detailed analysis regarding these points combined with the results of *exps 2, 3*, and *4*. Following the increase in pupil dilation at the introduction of the force field, the tonic baseline pupil diameter showed an increase in the following trial (Fig. 2*D*, black arrows) (*t*_27_ = 5.09, *P* = 2.4 × 10^−5^, two-sided paired *t* test), whereas it did not show such an increase at the removal of the perturbation (Fig. 2*D*, gray arrows) (*t*_27_ = 0.001, *P* = 0.99, two-sided paired *t* test). A two-way repeated-measures ANOVA also revealed the significant interaction between factors Trial and Phase (learning/washout) (*F*_1,27_ = 6.17, *P* = 0.02). Thus, the results seem consistent across the phasic pupil dilation and tonic baseline pupil diameter, including the weaker response at the removal of the force.

These findings appear to be consistent with recent proposals that task-induced pupil dilation reflects surprise and the tonic baseline pupil diameter reflects subjective uncertainty about the environment (15, 16). Theoretically, these variables can respond differently to the same error depending on participants’ knowledge about the task. For example, if participants had experienced large errors in a task, a similar error observed next time would be less surprising in the same task. Similarly, if participants knew that the environment switched to a known state, subjective uncertainty about the current environment should be small.

The presence of such knowledge can be tested by comparing the speed of adaptation and deadaptation. Faster deadaptation can be taken as evidence that the participants were aware that the force field was off and could use this knowledge to readjust their motor command. As expected, both the speed of error reduction and change in learning index were faster for the deadaptation (i.e., washout) phase. The participants showed systematically smaller number of trials to achieve either 40, 50, 60, 70, or 80% performance change for deadaptation than for adaptation (Supplemental Fig. S2, *C–F*; *F*_1,26,86_ = 4.45, *P* = 0.044 for lateral deviation, *F*_1,26.96_ = 11.45, *P* = 0.002 for learning index, assessed by mixed-effects model ANOVA). For a complementary analysis, we fitted the two-state model (51) to the learning index data excluding the washout data from the training data and predicted how fast learning index returns to the baseline level at the washout phase. Consistent with the previous analysis, the actual learning index data showed systematically smaller learning indices than predicted data (Supplemental Fig. S2*B*; test data). Notably, the test data fell below the lower 95% bound for five consecutive trials (2nd through 6th washout trials). The results thus suggest faster deadaptation even after assuming the two-state learning process and taking the forgetting to baseline into account. This indicates the participants’ use of some knowledge about the null-force field environment. Such knowledge may be associated with reduced pupil responses on the removal of the force field, although, at this point, fatigue or reduced arousal could also explain the results.

To further investigate the relationships between pupil diameter, error size, and environmental change during motor learning, we also monitored the pupil diameter in two additional experiments. In *exp 2*, we used a switching force field schedule, in which the direction of the force field was unexpectedly reversed multiple times, inducing overt environmental change and a reincrease in error. In *exps 3* and *4*, we used a gradual force field schedule, in which the magnitude of the force field was gradually increased, inducing covert environmental change with much smaller errors compared with *exps 1* and *2*.

### Pupil Responses to a Switching Force Field Schedule: Dissociation Between Error Sizes and Pupil Responses After Multiple Reversals

**Figure 3.**
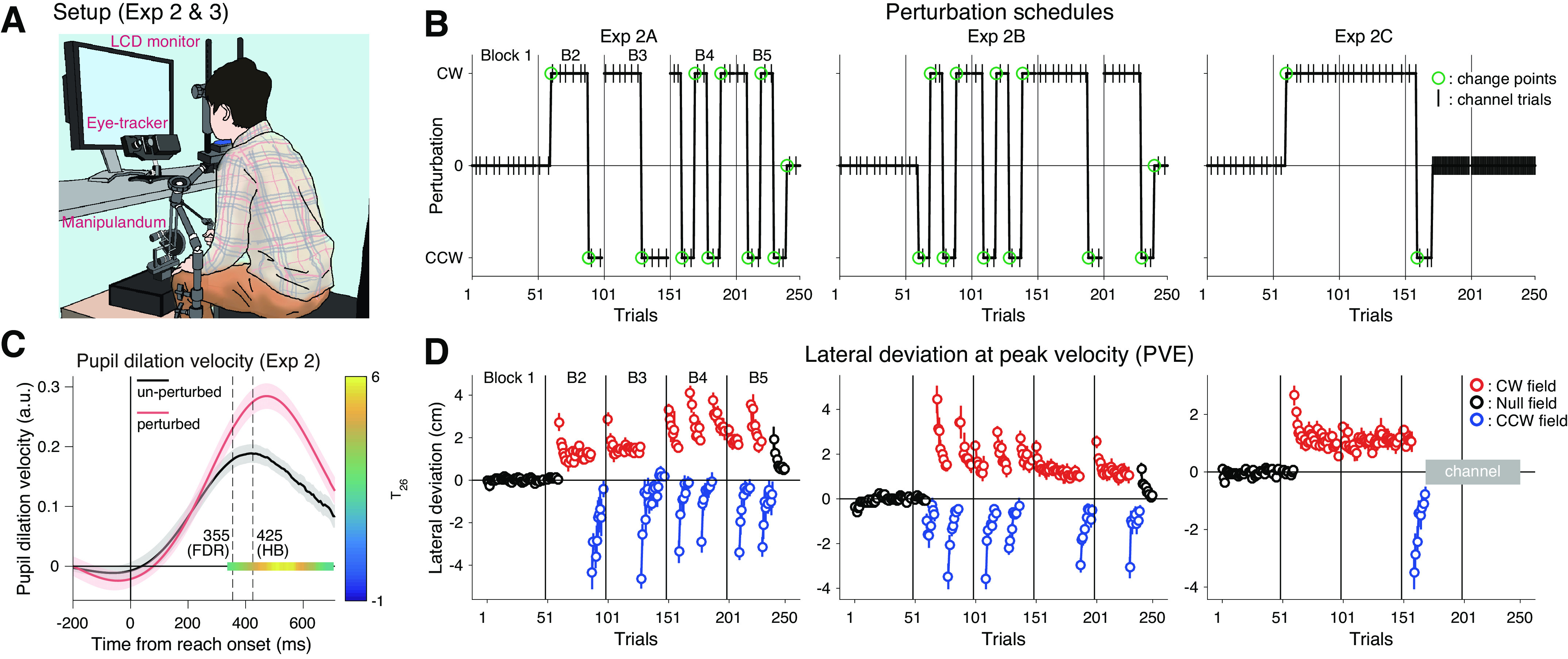
Pupil responses to sudden reversals in the force field. *A*: experimental setup for *experiments 2 and 3*. *B*: perturbation schedules for *exp 2* [*exp 2A* (*n* = 10 subjects), *2B* (*n* = 10 subjects), and *2C* (*n* = 9 subjects)]. The green circles indicate the change point trials in which either the magnitude (on/off) or direction (CW/CCW) of the force field changed in the middle of the blocks (changes across the blocks were excluded). The vertical lines indicate set breaks. *C*: the time course of the pupil dilation velocity averaged over the baseline trials (unperturbed; black trace) or the first five perturbation trials in the second block (perturbed; red trace), averaged over all subgroups. The areas around the traces represent SEM across the participants. The vertical dashed lines indicate the first time point of significant difference by running paired *t* tests (two-sided) corrected with either the Holm–Bonferroni method (HB; *P* < 0.05) or the Benjamini–Hochberg method (FDR; *q* < 0.05). A significant (*P* < 0.05) cluster assessed by cluster-based permutation test is shown as a color map on the *y* = 0 line representing *t* values for the comparison (T26 because of missing data; the color bar on the right). *D*: trial-by-trial change in the mean lateral hand deviation at the peak tangential handle velocity for *exp 2A* (first column), *2B* (second column), and *2C* (last column), respectively. The colors of circles (black, red, and blue) indicate the data for baseline, CW field, and CCW field, respectively. Positive values for *D* correspond to rightward deviation. The vertical lines indicate set breaks. Error bars represent SEM across participants. Trials in which the dot color changed indicate the change points. CCW, counter-clockwise; CW, clockwise; FDR, false discovery rate.

In *exp 2*, we monitored the pupil responses of another 29 participants while they reached in the presence of force fields with slightly different experimental setup compared with *exp 1* (i.e., a light-weight manipulandum and a vertically aligned monitor: see Fig. 3*A*). *Exp 2* involved three subgroups (*exps 2A*, *2B*, and *2C*) in which participants experienced different force field schedules (Fig. 3*B*). In all of the subgroups, the force field was abruptly introduced on the 11th trial in *block 2* (CW for *exps 2A* and *2C*, and CCW for *exp 2B*), and the direction of the force field was reversed at different timings and frequencies for different subgroups (Fig. 3*B*; see materials and methods for detail). These change points, including the introduction and removal of the force field, induced sudden, unexpected increases in error size (Fig. 3*D*), indicating a change in task environment. We examined participants’ pupil responses, both phasic and tonic, to these change points.

**Figure 4.**
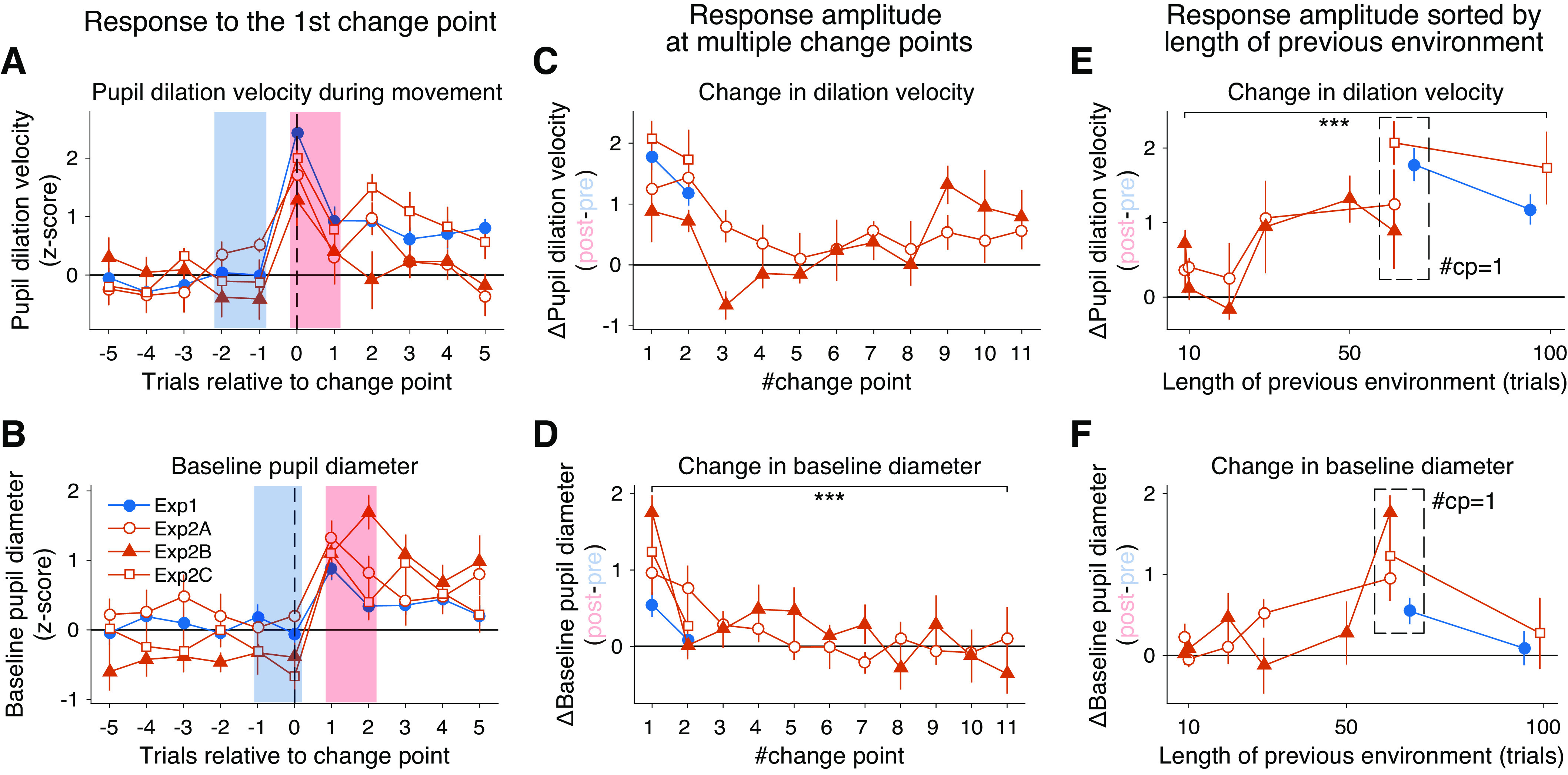
Pupil responses around change points. *A* and *B*: trial-by-trial change in the z-transformed mean pupil dilation velocity (*A*) and baseline pupil diameter (*B*) around the first change point. Pre- and postchange point values are defined by blue and red shading, respectively. *C* and *D*: the amplitude of change point-induced mean pupil dilation velocity change (*C*; difference between post- vs. prechange point values in *A*) and baseline pupil diameter change (*D*; difference between post- vs. prechange point values in *B*) as a function of the number of change points. *E* and *F*: the amplitude of change point-induced mean pupil dilation velocity (*E*) and baseline pupil diameter (*F*) changes as a function of the length of previous perturbation environment. The error bars indicate SEM across the participants [*exp 2A* (*n* = 10 subjects), *2B* (*n* = 10 subjects), and *2C* (*n* = 9 subjects)]. Asterisks indicate a significant slope for the #change point assessed by the linear mixed-effects model (***P < 0.001; see main text and materials and methods for more detail).

First, to replicate the basic results of pupil responses in *exp 1*, we analyzed the pupil data around the first change point (i.e., the first introduction of force field) aggregating the data from all subgroups. The time course of perturbation-evoked phasic pupil dilation showed a similar pattern (Fig. 3*C*) to that in the first experiment (Fig. 2*B*) with a slight difference in the timing of the effect. Thus, we used a similar time window for averaging the pupil dilation velocity at each trial (300–700 ms). As shown in Fig. 4, *A* and *B*, both the phasic pupil dilation during movement and the tonic baseline pupil diameter responded to the first change point in a similar way to that in *exp 1*. How does the pupil respond to the following change points?

Intriguingly, across subsequent change points (Fig. 3*D*), both the pupil dilation and baseline pupil diameter quickly became insensitive to the large induced movement errors (Supplemental Fig. S3), indicating that pupil does not simply reflect the movement error itself. It should be noted that such a decline in pupil response sensitivity to errors was not reported previously in the context of reinforcement learning (e.g., Refs. 14, 15). To quantify the decline in pupil responses, we focused on response amplitudes around each change point. The response amplitude in the pupil dilation and the baseline pupil diameter showed a dramatic decline for the subsequent change points (Fig. 4, *C* and *D*). Such reduction of pupil responses was surprising given the almost twofold increase in the size of the unsigned error at the second change point because of the reversal of force field direction (Fig. 3*D*). The observed insensitivity to error is unlikely to have been caused by fatigue or boredom alone, as participants in *exp 2B* already showed a substantial decline in both pupil dilation and baseline diameter changes at the third and second change point, respectively (Fig. 4, *C* and *D*), which was still in the second block of the experiment (80th and 70th trial).

In parallel with the reduced pupil response over the multiple change points, visual inspection of the data suggested that there was a sudden increase in the phasic pupil response on the 9th change point in *exp 2B*, which was after the constant perturbation period (Fig. 4*C*). Given this effect, we also replotted the pupil responses at each change point as a function of the length of its previous perturbation period (Fig. 4, *E* and *F*). The resultant plots showed that the pupil dilation had a positive relationship with the perturbation length, indicating that pupil dilation reacts more to the changes after experiencing a long, stable environment (Fig. 4*E*). A linear mixed-effects model on the pupil dilation revealed a significant effect of the length of previous environment (*t*_174_ = 4.33, *P* = 2.51 × 10^−5^), while no significant effect of the number of change point (*t*_174_ = 1.38, *P* = 0.17), after controlling for the random group/individual factors and the effect of trajectory errors at each change point (see materials and methods). Interestingly, a similar mixed-effects model on the baseline pupil diameter revealed the opposite; the significant effect of the number of change point (*t*_233_ = −4.49, *P* = 1.14 × 10^−5^), while no significant effect of the previous environmental length (*t*_233_ = 1.91, *P* = 0.06). These results suggest that the pupil responds to errors, taking either environmental statistics (for phasic dilation) or novelty (for tonic baseline diameter) into account.

The rapid change in pupil response sensitivity to movement errors in repeated change points, including the results of *exp 1*, implies some form of cognitive learning going in parallel with motor learning. This may include implicit/explicit knowledge about the task (e.g., the presence of change points, the force to the handle, or contextual boundary), which could lead to better motor performance when encountering the same perturbation, a phenomenon known as “savings” (58–60). A recent line of studies proposed that the savings in force field learning are manifested as sophistication in online feedback control (feedback adaptation), which is characterized by more adapted performance (e.g., smaller trajectory error) at the very first trial of reexposure (61, 62). Consistent with this view, when we looked closely at the kinematic errors at the change point trials (i.e., the exact trial of force reversals), we saw that participants gradually became better at handling sudden changes in mechanical perturbations. The size of trajectory errors at the point of peak velocity (PVE) for the change point trial 2 through 10 (i.e., unexpected reversal in force field) exhibited a systematic reduction as participants experienced more reversals (Supplemental Fig. S4*A*; *t*_177_ = −3.91, *P* = 1.31 × 10^−4^, a significant negative slope for change point number assessed by a linear mixed-effects model). Such a reduction in PVE was not simply caused by a similar reduction of peak y-velocity in these change point trials (tendency toward increase: *t*_175_ = 1.94, *P* = 0.054, slope assessed by a linear mixed-effects model; Supplemental Fig. S4*C*) or a systematic reduction in learning in feed-forward motor commands in the previous force field environment (no systematic decline in learning index measured at the closest channel trial before each change point: *t*_193_ = −0.43, *P* = 0.67, linear mixed-effects model; Supplemental Fig. S4*B*). Note that, as already mentioned, the decrease in pupil responses was significant even after taking this reduction of kinematic errors into account. Our data, thus, showed some consistency with the recently proposed signature of savings in force field learning.

### Pupil Responses to Gradual Force Perturbation: Association Between Baseline Pupil Diameter and Incidental Knowledge of Perturbation

**Figure 5.**
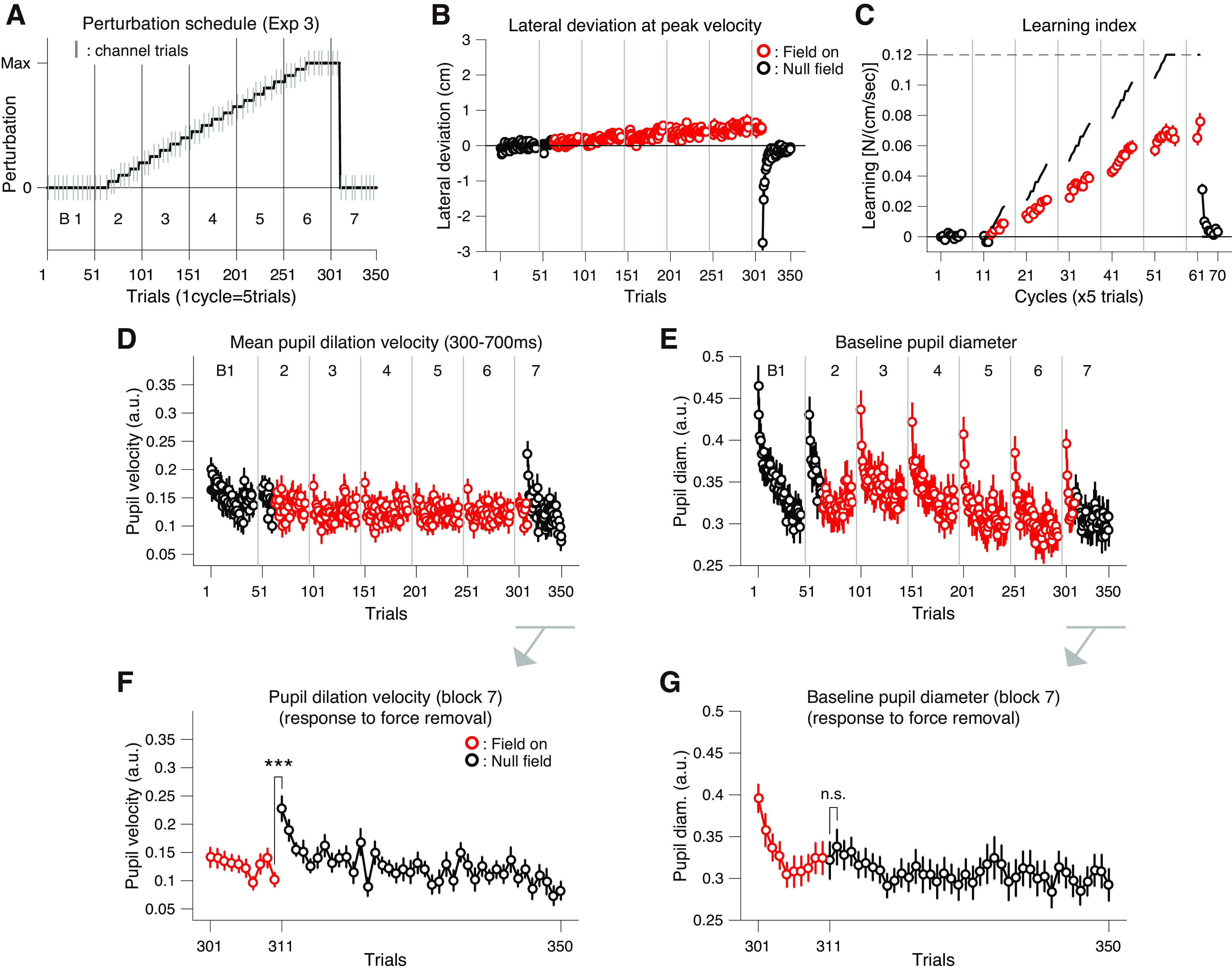
Pupil responses to gradually introduced force field (*experiment 3*). *A*: the perturbation schedule for *exp 3* (*n* = 30 subjects). The size of the force field was gradually increased with small steps (see materials and methods). The vertical lines indicate set breaks. The short gray vertical lines represent the force channel trials. *B*: mean lateral hand deviation at the peak tangential handle velocity for each trial. Positive values correspond to rightward deviation. *C*: mean learning index measured in the channel trials (once in a cycle). Learning index was the lateral force to the channel at the time of peak velocity divided by the peak velocity (i.e., viscosity). Solid lines above the plots indicate ideal values for the learning index (*A*) averaged within each cycle. *D*: mean pupil dilation velocity (averaged between 300 and 700 ms since the movement onset) for each trial. *E*: the baseline pupil diameter for each trial. *F* and *G*: mean pupil dilation velocity data (*F*) and the baseline pupil diameter data (*G*) around the point of force removal in the *block 7*. The red dots represent values for perturbation trials (*B–G*). Error bars correspond to SEM across participants (*B–G*). The asterisks indicate statistical significance (***P < 0.001).

We also ran another experiment with 30 new participants in which they adapted to a gradually introduced force field (Fig. 5*A*; see materials and methods for details). As previously noted, the gradual introduction of perturbations is believed to evoke substantially less awareness about the presence of perturbation compared with abruptly introduced perturbations because of the smaller errors it induces (63, 64). As expected, the magnitude of error experienced by participants was much smaller compared with *exps 1* and *2* (Fig. 5*B*). As shown in Fig. 5*C*, participants gradually adapted to the force field to a level that was comparable to that shown by participants in *exp 2C* (Supplemental Fig. S3*B*). There was no significant difference in learning indices between the average of the last five channel trials in the CW field for *exp 2C* versus the average of the last five channel trials before the start of constant channel phase for *exp 3* (*t*_37_ = −1.25, *P* = 0.22, two-sided independent samples *t* test). How does the pupil respond to gradual perturbation?

As expected, except for the clear and consistent increase in baseline pupil diameter at the start of new block, participants’ pupils showed no clear responses to the gradually increasing force field, in terms of both phasic and tonic activity (Fig. 5, *D* and *E*). The results clearly indicate that the pupil responses were more sensitive to a sudden and large change in the environment (*exps 1* and *2*) than to a covert, gradual change (*exp 3*). Notably, the pupil dilation during movement showed a sharp rise in response to the sudden increase in error caused by the removal of the force field (*t*_25_ = 4.87, *P* = 5.80 × 10^−5^; Fig. 5*F*), whereas the baseline pupil diameter did not (*t*_30_ = 1.03, *P* = 0.31; Fig. 5*G*). This is consistent with what we observed for *exp 1*; the removal of force field only elicited the phasic pupil dilation (Fig. 2, *C* and *D*). Thus, so far, the results from *exps 1–3* are consistent in that the phasic pupil dilation during movement may reflect surprise about movement errors (not errors per se) and the baseline pupil diameter may reflect subjective uncertainty and/or unfamiliarity about the environment.

**Figure 6.**
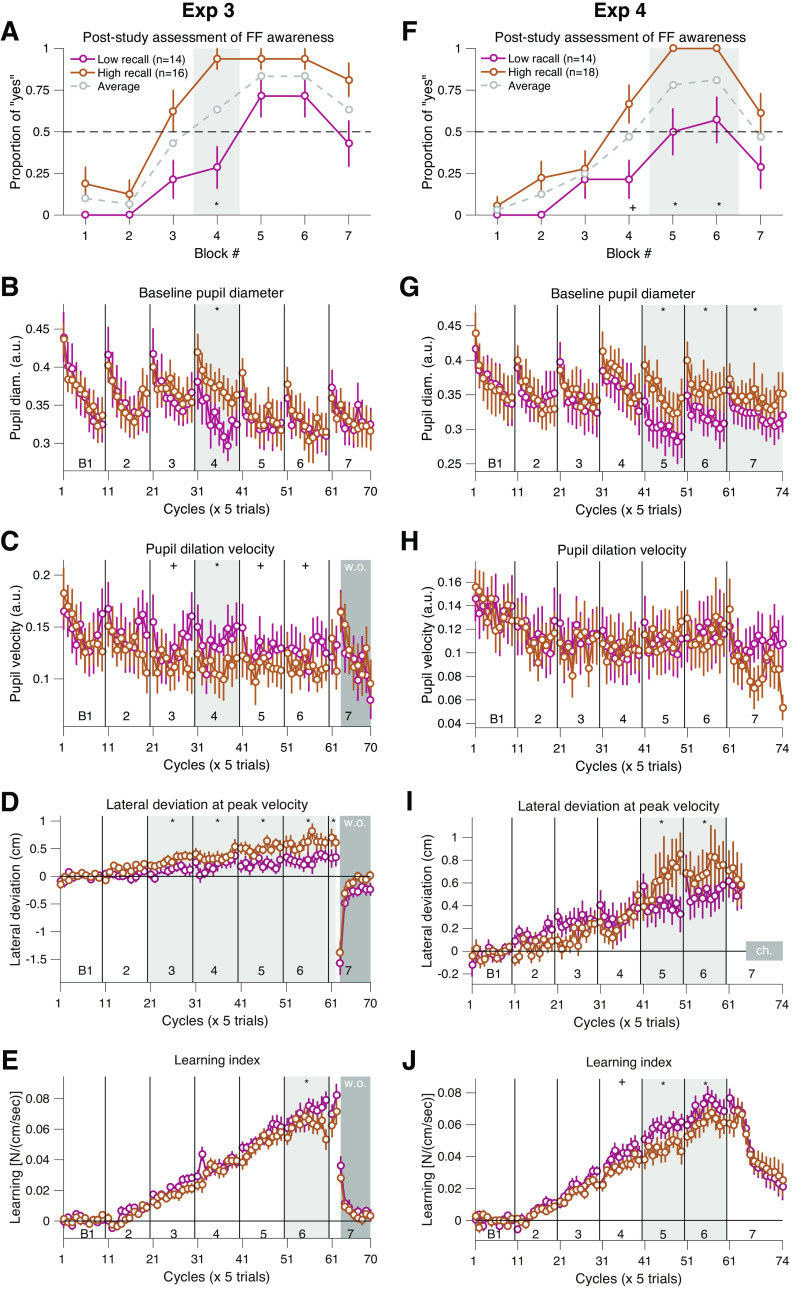
Individual differences in baseline pupil diameter were correlated with later recall of the force field (*experiments 3 and 4*). *A* and *F*: the score of the poststudy questionnaire for awareness of the force field perturbation for each block for *exps 3* and *4*. Data were split into two subgroups according to the median of the total score (number of “yes” responses) over the blocks (below-median: purple dots, above-median: gold dots). Gray dots indicate the mean score for all participants. *B–E, G–J*: median split data for baseline pupil diameter (*B* and *G*), pupil dilation (*C* and *H*), PVE (*D* and *I*), and learning index (*E* and *J*). Data were averaged within each cycle (five-trial bins). Error bars correspond to SEM across the participants. Areas shaded by light gray indicate a significant difference (*χ*^2^ test for *A* and *F*; linear mixed-effects model for *B–E, G–J*). The data indicated by dark gray shades (w.o., wash out for *C–E*, and ch., channel for *I*) were excluded from the statistical analysis. *Significant effect after correcting for multiple tests using the Holm–Bonferroni method controlling for family-wise error rate (*P* = 0.05). +Significance assessed using the Benjamini–Hochberg method controlling for false discovery rate (*q* = 0.05). Raw *P* values are reported in the main text. The overall group difference existing already in *block 1* was subtracted for visualization purpose. PVE, peak velocity error.

We then asked to what extent the pupil responses reflect the individual difference in cognitive/motor learning behaviors. Compared with the obvious changes in the force environment in *exps 1* and *2*, the gradual force introduction in *exp 3* would be more suitable for this purpose. As one such measure, we asked the participants to report the presence/absence of force perturbation for each block immediately after the experiment (not during the task). This was to avoid inducing any potential cognitive bias, and the participants were also not informed about this interview before the main session (see materials and methods for more detail). If one had perfect sensitivity and memory, he/she would answer “no” for the *block 1* and “yes” for the rest of the blocks (i.e., [0, 1, 1, 1, 1, 1, 1]). The average report data showed a gradual increase in report rate, consistent with the gradual increase in the force field (Fig. 6*A*). Using this report score, we split the participants into two subgroups according to the median of total number of “yes” report (for more detail, see materials and methods). This yielded two subgroups of 16 above-median participants (“High-recall”) and 14 below-median participants (“Low-recall”) (Fig. 6*A*). Note that the unequal number for the subgroups is due to the facts that *1*) the total report score took discrete values (0–7) and *2*) the rank order of participants contained ties. The *χ*^2^ test revealed a significant difference in the report rate between the subgroups in block #4 (*χ*^2^ = 13.66, *P* = 2.19 × 10^−4^; Fig. 6*A*). There was no significant bias in applied force direction between the subgroups (*χ*^2^ = 2.14; *P* = 0.14), indicating that the difference was not simply caused by the different sensitivity to force direction. We then compared the pupillary and behavioral measures between the subgroups.

Interestingly, comparison of pupil data between subgroups revealed that the baseline pupil diameter was significantly larger in the same *block 4* for the “High-recall” participants (*F*_1,2022_ = 26, *P* = 3.70 × 10^−7^; Fig. 6*B*), detected by a significant subgroup × block interaction terms used by a linear mixed-effects model (see materials and methods for more detail). Remarkably, applying the same split analysis to the data for another set of participants who also learned a gradual force field (*exp 4*; for details, see Supplemental Fig. S5) showed similar results. The split revealed significant differences in the report rate between the subgroups in *blocks 4* (*χ*^2^ = 6.47, *P* = 0.01), *5* (*χ*^2^ = 11.52, *P* = 6.90 × 10^−4^), and *6* (*χ*^2^ = 9.49, *P* = 0.002) (Fig. 6*F*). Consistently, the baseline pupil diameter was larger for the “High-recall” participants in the same *blocks 5* (*F*_1,2286.5_ = 26.57, *P* = 2.75 × 10^−7^), *6* (*F*_1,2286.5_ = 19.25, *P* = 1.20 × 10^−5^), and *7* (*F*_1,2286.5_ = 7.48, *P* = 0.006) (Fig. 6*G*). These results suggest some association between retrospective judgment of perturbation presence and the baseline pupil diameter during the task. Curiously, compared with the robust association between the recall of perturbation and baseline pupil diameter, the pupil dilation showed a weaker association with the recall. Although the pupil dilation of the “High-recall” participants was smaller in the *block 4* (same as in the report data) in *exp 3* (*F*_1,1782.5_ = 12.32, *P* = 4.60 × 10^−4^; Fig. 6*C*), no group difference was detected in *exp 4* (Fig. 6*H*).

For movement error and learning index, the results of the two experiments were consistent in that the participants with higher perturbation recall experienced more error and showed reduced learning. For *exp 3* data, movement error was significantly larger for the “High-recall” participants from *block 3* through *7* (*F*_1,1782.5_ > 8.11, *P* < 0.0044; Fig. 6*D*). In contrast, “High-recall” participants showed smaller learning index in *block 6* (*F*_1,1782.2_ = 11.36, *P* = 7.68 × 10^−4^; Fig. 6*E*). For *exp 4* data, comparison revealed larger movement error in *blocks 5* (*F*_1,1971.5_ = 10.64, *P* = 0.001) and *6* (*F*_1,1971.5_ = 8.62, *P* = 0.003), as well as smaller learning index values in *blocks 4* (*F*_1,2286.1_ = 5.31, *P* = 0.02), *5* (*F*_1,2286.1_ = 19.75, *P* = 9.24 × 10^−6^), and *6* (*F*_1,2286.1_ = 9.79, *P* = 0.0017) in the “High-recall” participants (Fig. 6, *I* and *J*). Overall, the results suggest some interesting links between retrospective judgment or incidental memory of the perturbation, baseline pupil diameter, and motor learning.

### Increased Baseline Pupil Diameter at the Start of New Blocks Suggests Increased Subjective Uncertainty

**Figure 7.**
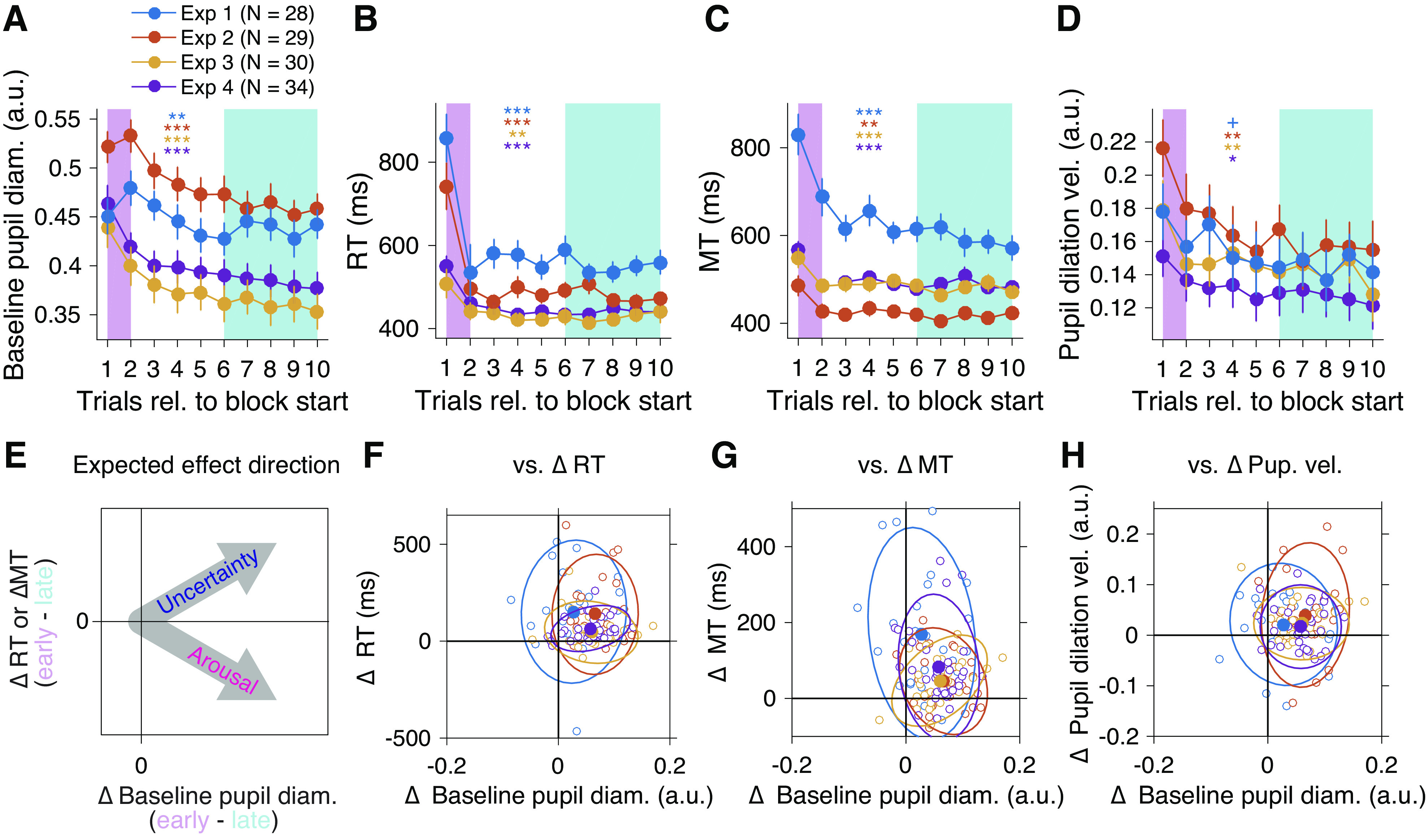
Dilated baseline pupil diameter at the start of a block implies increased subjective uncertainty (*experiments 1–4*). Change in baseline pupil diameter (*A*), pupil dilation velocity (*B*), reaction time (*C*), and movement time (*D*) for the first 10 trials after the set break, averaged over multiple blocks (see materials and methods). Error bars indicate SEM across participants. *E*: a schematic illustration of the association between the delta-baseline pupil diameter and delta-RT or -MT expected either from uncertainty or an arousal/vigilance account of baseline pupil diameter (arrows). Scatter plot for delta-baseline pupil diameter vs. delta-RT (*F*), delta-MT (*G*), or delta-pupil dilation velocity (*H*), calculated as the difference between averages indicated by colored areas (pink: early trials, cyan: late trials) in *A–D*. In all cases (*F, G*, and *H*), the group-level effect lies in the first quadrant, consistent with the uncertainty account of increased baseline pupil diameter illustrated in *E*. Solid closed dots represent averaged data. Open dots represent individual data. Ellipses indicate the 95% confidence contour. Asterisks (colors correspond to experiment as indicated in *A*) indicate significance assessed by two-sided paired *t* test between the averaged data for early vs. late trials (+*P* < 0.1, *P < 0.05, **P < 0.01, ***P < 0.001). Note that although we plotted the data from the four experiments together, due to the different settings across the experiments (movement amplitude, manipulandum weight, pupil normalization) direct across-experiment comparison would not be feasible.

The most prominent feature for the pupil responses in *exp 3* was a characteristic reincrease in baseline pupil diameter at the beginning of a new block (Fig. 5*E*). This phenomenon was robustly accompanied by extended reaction time (RT) and movement time (MT). Interestingly, these features were also consistently observed in *exps 1, 2*, and *4*. Analyzing the data from all of the experiments revealed a consistent pattern of modulation between baseline pupil diameter, RT, and MT, all of which showed larger values in earlier trials in the block (Fig. 7, *A*–*C*) (see materials and methods for detail). The comparison between the average of early versus late trials consistently yielded significant differences between them (statistics summarized in the Table 2). Notably, if the increased tonic baseline pupil diameter at the start of the new block merely indicated higher arousal/vigilance/vigor because of short rests between the blocks, the RT and MT would be expected to decrease (Fig. 7*E*) (28, 29, 55, 65, 66). However, they showed the opposite (Fig. 7, *F* and *G*), suggesting another interpretation.

**Table 2.** Statistical values for the comparison between early vs. late trials after set breaks (Fig. 7*)*

Experiments	Variables	*t* Values	df	*p*-Values
*Exp 1*	Baseline pupil diameter	3.06	27	0.005
	Mean pupil dilation velocity	1.83		0.078
	Reaction time	4.27		2.13 × 10^−4^
	Movement time	6.34		8.58 × 10^−7^
*Exp 2*	Baseline pupil diameter	8.99	28	9.57 × 10^−10^
	Mean pupil dilation velocity	2.95		0.006
	Reaction time	4.70		6.23 × 10^−5^
	Movement time	3.41		0.002
*Exp 3*	Baseline pupil diameter	7.56	29	2.47 × 10^−8^
	Mean pupil dilation velocity	3.43		0.0018
	Reaction time	3.10		0.0043
	Movement time	4.19		2.37 × 10^−4^
*Exp 4*	Baseline pupil diameter	9.26	33	1.07 × 10^−10^
	Mean pupil dilation velocity	2.34		0.026
	Reaction time	6.29		4.13 × 10^−7^
	Movement time	4.88		2.64 × 10^−5^

Two-sided paired *t* tests were used for the comparison.

Longer RTs typically accompany decisions under uncertain condition or choices with many potential options (Hick’s law), and longer MTs are observed when the task demands higher accuracy (speed-accuracy trade-off). Here, the longer MT can be interpreted as increase in internal criteria for accuracy due to increased uncertainty. Therefore, such increases in baseline pupil diameter after set breaks could, at least in part, reflect an internal state related to increased subjective uncertainty. The consistency of the pattern further supports the argument that the baseline pupil diameter reflects subjective uncertainty about the environment.

The pupil dilation velocity also showed a similar pattern (Fig. 7, *D* and *H*), suggesting that the similar sensory outcomes^1^ were more surprising (or informative) under higher subjective uncertainty about the task environment. Conversely, this indicates that the similar sensory outcomes would be less surprising when uncertainty about the environment is low. Thus, the result also seems consistent with reduced phasic pupil dilation at washout (*exp 1*) or frequent switching of force field directions (*exp 2*), where the uncertainty was presumably low.

## DISCUSSION

In the current study, we systematically studied pupillary responses during the process of force field reach adaptation in which the perturbation was applied either abruptly (*exp 1*), abruptly with multiple reversals of the force direction (*exp 2*), or gradually (*exps 3* and *4*). The results can be summarized as follows: *1*) unexpectedly large movement error led to increased phasic pupil dilation during movement that was accompanied by a transient increase in tonic baseline pupil diameter in subsequent trials (*exps 1–4*); *2*) such pupil responses to error were attenuated when participants repeatedly experienced large errors induced by multiple change points (*exps 1* and *2*) or when the error was due to the environmental change to known condition (i.e., null force) (*exps 1* and *3*); *3*) baseline pupil diameter showed consistently larger values at the beginning of each experimental block, which were consistently accompanied by increased reaction time and movement time (*exps 1–4*); and *4*) later recall of gradually introduced force field correlated with baseline pupil diameter during the task (*exps 3* and *4*). These results constitute the first detailed characterization of pupillary responses during human motor learning.

As will be discussed in the following section, our results support an interpretation that is generally consistent with proposals in the field of cognitive, reward-based learning (13–19, 30–32), suggesting that baseline pupil diameter is likely to reflect subjective uncertainty about the environment, whereas phasic pupil dilation during movement is likely to reflect surprise about sensory consequences (e.g., movement error), and further suggest that both pupil responses are modulated by the novelty of the environment. When the novelty of the environment is high, such as at the beginning of a new block (i.e., participants were unsure about what will happen next or were unsure of the experimental manipulation from the previous block) or at the introduction of unknown force field, both the tonic baseline pupil diameter and phasic pupil dilation were high. In contrast, when the novelty of environment is low, such as at removal of force field, or at change points in which the force direction is switching between known states, pupil responses are less sensitive to the size of the observed error. As we discuss in the following, these processes may, at least in part, be mediated by central noradrenaline (NA) activity.

### What Do Pupil Responses Reflect During Motor Adaptation?

Neurophysiologically, pupil diameter under constant luminance is known to reflect the activity of the locus coeruleus (LC) in the brainstem, a central source of NA (53, 67, 68). As suggested by numerous studies, pupil size and LC neurons show similar response patterns to surprising events and perceived environmental uncertainty (53, 67). For instance, both LC neurons and the pupil show phasic responses to novel, infrequent, or surprising stimuli (14–19, 69–72). Moreover, LC neurons and the pupil show a transient increase in tonic activity facing the reversal of target-reward contingency (i.e., sudden increases in prediction error) in both monkeys (70) and humans (13–18, 30–32). Thus, phasic pupil dilations and tonic increases in baseline pupil diameter are likely to reflect perceived surprise and environmental uncertainty, respectively, mediated by corresponding activation in the LC-NA system. Thus, the present results can be interpreted as follows. A sudden introduction or reversal of the force field induced substantial surprise, leading to a phasic response in the pupil-linked NA/arousal system. This is then followed by a transient increase in uncertainty regarding the task environment and a corresponding increase in tonic activity of the pupil-linked NA/arousal system (*exps 1* and *2*). The absence of such pupil responses in the gradually increasing force field condition (*exps 3* and *4*) suggests that the size of (prediction) error is a key (but not only, as we discuss later) factor driving this process.

Interestingly, further observation indicated that pupil responses to errors might also be modulated by participants’ experience, such as novelty of perturbation and/or recent history of error size (i.e., length of perturbation schedule). The decline in pupil responses to large errors at the removal of the force field (*exp 1*; Fig. 2, *C* and *D* and Supplemental Fig. S2*A*) and at multiple change points (*exp 2*; Fig. 4, *B* and *D*) indicates that error size is not the only determinant of pupil responses during motor adaptation. Conceivably, knowledge about the task (e.g., the presence of reversal) might have made the sudden increase in movement errors no longer surprising, but somehow expected. A recent monkey study using a choice-reversal task also reported that the more reversals monkeys experienced, the faster they switched behavior, which was captured by a Bayesian choice model as gradually increasing prior belief on reversal (73). Such faster readaptation after experiencing single/multiple perturbations has been reported also in the context of savings (58–60) or structural learning (74–76). In accord with this notion, we observed a faster deadaptation (*exp 1*) and a gradual reduction in kinematic error in the change point trials (*exp 2*), which could be because of increased stiffness (77, 78), improved feedback control (61, 62), or both. It should be noted that the decline in pupil sensitivity to frequent increases in errors, as observed in the present study, has not been described for a similar reversal learning paradigm in the context of reward-based learning (e.g., Refs. 14, 15). Although it is not yet clear whether this phenomenon is specific to motor learning, our results extend the findings of previous studies in revealing how the pupil-linked arousal/NA system responds to changes in the environment.

We speculate that the larger baseline pupil diameter observed at the beginning of new experimental blocks reflects heightened subjective uncertainty about the task (e.g., Fig. 5*E*; *exp 3*). This characteristic pattern of larger pupil size early in experimental blocks has also been frequently reported but often interpreted simply as (re-)increase in arousal after short rests (e.g., Refs. 68, 69). However, as indicated by the increased RT and MT in this phase (Fig. 7), the larger pupil diameter here was more likely to be associated with increased subjective uncertainty because RT typically increases when facing uncertain decisions (79) or choices with many potential options (80), and MT typically increases when the task is difficult (i.e., higher accuracy demand) (81). Although the physical difficulty of the current task (target size and distance) was kept constant, the longer MT can be interpreted as increased subjective difficulty, such as increased internal accuracy demand or decreased internal estimate of self-movement accuracy. A previous study has reported that the participants can choose optimal movement time to maximize performance gain (e.g., task success, monetary reword, etc.) taking their own speed-accuracy trade-off function into account (82). A recent study also reported a larger pupil diameter and slower RT for uncertain decisions (52). If larger baseline pupil diameter only reflects higher arousal/wakefulness (24–27), vigilance/alertness (28, 29), and/or vigor (65, 66, 83), RT and MT would be expected to decrease (Fig. 7*E*). Thus, our data suggest that larger pupil diameter after set breaks at least partially reflects subjective environmental uncertainty.

### How Can Pupil-Linked Arousal Influence Motor Adaptation?

The transient increase in subjective uncertainty in the environment and putative pupil-linked arousal/NA activity induced by the sudden introduction of force fields bears a similarity to several phenomena reported in the early phase of human motor adaptation. For example, adaptation to both stable and unstable force fields transiently increases muscle coactivation (78, 84, 85), as increasing limb impedance is an optimal strategy for movement under highly uncertain dynamic environments (77) or to increase movement accuracy (86). Similarly, adaptation to a force field transiently increases the gains of visuomotor feedback responses (87), cortically mediated long-latency muscle stretch reflex (88), and motor-evoked potentials induced by single-pulse transcranial magnetic stimulation (89), the latter two of which are mediated by the primary motor cortex (90–92). Furthermore, a recent study demonstrated that the Ia afferent firing from muscle spindles is enhanced in the early phase of visuomotor adaptation (93). These central and peripheral gain control changes might originate from the NA projections to the motor cortices and spinal cord (94). Notably, a recent report showed an arousal-like transient increase in neuronal population activity in the primary motor cortex in response to errors caused by environmental (brain-computer interface mapping) change, which correlated with pupil diameter change (95). Additional evidence suggests increased corticospinal excitability (96) and muscle spindle sensitivity (97, 98) following sympathetic upregulation.

The association between the interindividual differences in the retrospective report rate of perturbation and the baseline pupil diameter as well as the amount of learning and movement error (Fig. 6) may suggest potential influence of the pupil-linked arousal/NA system on memory of perturbation and implicit adaptation^2^. Although the causal relationship among these variables should be complex, one possible interpretation would be that participants in the “High-recall” group experienced somehow higher subjective uncertainty about the task that might have led to more frequent exploratory behavior (30, 31, 53) and resulted in a smaller amount of adaptation in desired direction and larger errors. This scenario is consistent with previous reports that the awareness of perturbation reduced the amount of implicit adaptation (64, 100–102) and more recent studies suggesting competition between the implicit adaptation process and explicit strategy (103, 104). The memory of higher subjective uncertainty and/or possible exploration may have increased the probability of reporting perturbation presence later in the interview. It is also well established that the NA enhances declarative memory, especially under emotional/stressful context (105, 106).

Although there was a robust association between the report rate and the baseline pupil diameter, the phasic pupil dilation showed inconsistent results (Fig. 6, *C* and *H*). This is presumably because the errors and their group differences were small given the use of gradual force field. This leads to another interpretation regarding larger error (and smaller adaptation) as the cause of larger baseline pupil diameter and an increased report rate to be less likely. Nevertheless, it is still possible that the small differences in error-induced surprise that were undetectable by pupil measure might have accumulated into detectable differences in the baseline pupil diameter. Overall, although we certainly need more confirmatory studies, the current result suggests that the link between subjective experience of perturbation, pupil-linked arousal/NA activity, and motor learning warrants further investigation.

### Limitations and Open Questions

One topic that remains to be addressed is the relationship between motor learning rate and uncertainty/surprise and the pupil-linked arousal/NA system. Theoretically, statistically optimal learning algorithms, such as the Kalman filter (107) and more recent extensions (34–36, 108–110), take multiple sources of uncertainty into account to dynamically modulate the learning rate. Unfortunately, it is not possible to directly address this question with the current data, because the current experiments were not designed for the accurate quantification of learning rate. One recent study (111), however, reported an association between baseline pupil diameter and learning rate in a modified saccade adaptation task. We will address this question in a separate report in which we directly measure the single-trial learning rate with/without experimental manipulation of the pupil-linked arousal system in the motor adaptation paradigm.

Another intriguing future question is whether/how pupil responses can be informative for understanding the “explicit” and “implicit” components of motor learning (5, 6, 112). Currently, the dominant approach for trial-by-trial assessment of the conscious/explicit component of motor learning in the reach adaptation paradigm is to ask participants to verbally/manually report their aiming direction before each reach (5, 103, 113). However, this approach suffers from the inherent problem of interfering in the learning system itself and hence biasing learning processes to be more “explicit” (114–116). Moreover, the additivity assumption underlying this approach has recently been questioned (117). In addition, an accurate measurement of each component of learning does not necessarily provide direct clues regarding the underlying cognitive processes. Thus, it is important to develop new complementary approaches to independently assess the explicit and/or implicit component (e.g., 132, 133) and to also examine underlying cognitive states without affecting the learning system. Further research on pupil responses during motor learning may provide a window for understanding how learning about movement selection (i.e., explicit process) progresses.

Several different lines of computational framework have been proposed to understand the motor learning process, including explicit/implicit processes and apparent change in error sensitivity in motor learning. These frameworks include, for example, a multistate (usually two) state-space model consisting of fast and slow (51) or explicit and implicit processes (118), a class of model that uses consistency of error to modulate error sensitivity (119–121), and recently proposed contextual inference model (122). Although it is not the scope of the current study to identify under which framework the pupil measures can be most informative, we would note here several points. There have been a number of studies outside the context of motor control/learning that connect pupil responses to contextual inference. Recent evidence suggests that prediction error (123, 124), as well as phasic pupil responses (54, 125), can signal a subjective belief about environmental change, which helps to create event boundaries in a memory structure (54, 124). Similarly, a study has reported that the contextual boundary induces pupil dilation and facilitates later recollection (126). Although these studies may suggest pupil measures may have slightly more affinity to the contextual inference account of motor learning, further studies would be needed to clarify this issue.

Finally, despite the established link between pupil diameter and central LC-NA activity, the relationship is not necessarily one-to-one. For instance, a recent rodent study, in which cortical axons for both NA and acetylcholine (ACh) were recorded, reported that while rapid changes in pupil diameter and its time derivative (i.e., dilation velocity) were more strongly correlated with NA than ACh activity, slow pupil dilations on the timescale of a few seconds were correlated with activation of both NA and ACh (127). However, the detailed mechanisms by which central ACh affects pupil size remain unknown. Moreover, recent studies have reported that the activity of the dorsal raphe nucleus (DRN) serotonin (5-HT) neurons in mice also tracks environmental uncertainty (128) and that photoactivation of DRN 5-HT neurons also elicits pupil dilation (129). However, the bidirectional connection between DRN and LC (94) further complicates this relationship. It is also important to note that 5-HT plays a key role in controlling the input-output gain of spinal motoneurons (130, 131). Overall, a more direct approach, such as invasive animal studies or pharmacological manipulation, is required to further establish the links among pupil diameter, these neuromodulators (NA, ACh, and 5-HT), and motor learning processes.

In the present study, we provided the first detailed characterization of pupillary responses in a widely used motor adaptation paradigm. Our data revealed how the internal states of human participants, most likely surprise and uncertainty about the environment, dynamically change during motor adaptation, thus providing important clues for understanding the process of force field learning. The results of the current study highlight the utility of pupil diameter as a valuable window into the motor system.

## DATA AVAILABILITY

The data and the custom-written Matlab codes used for the analysis are available at https://github.com/ayokoi/pupilMotor01.

## SUPPLEMENTAL DATA

10.6084/m9.figshare.20742091.v1 Supplementary Note and Supplemental Figs. S1–S5: https://doi.org/10.6084/m9.figshare.20742091.v1.

## GRANTS

The present work is supported by JSPS Fellowships for Young Scientists (#15J03233) and KAKENHI (#18K17916 and #22H03501; to A.Y.), and postdoctoral fellowships from NSERC and the BrainsCAN program at Western University (to J.W.).

## DISCLOSURES

No conflicts of interest, financial or otherwise, are declared by the authors.

## AUTHOR CONTRIBUTIONS

A.Y. conceived and designed research; A.Y. and J.W. performed experiments; A.Y. analyzed data; A.Y. and J.W. interpreted results of experiments; A.Y. prepared figures; A.Y. drafted manuscript; A.Y. and J.W. edited and revised manuscript; A.Y. and J.W. approved final version of manuscript.

## Footnotes

1 Note that we excluded the trials with strong force field from this analysis (see Methods for more detail).

2 We assume the learning index assessed in *exps 3* and *4* more likely reflect implicit learning, as the perturbation was gradually introduced (63, 64). We also assume our instruction to participants for aiming straight at the target was effective throughout the task. This assumption is supported by a recent study reporting that single instruction before learning was as effective as multiple instructions during learning on the total learning (99).
